# Exome sequencing enables molecular diagnosis in 10% of early-onset or familial systemic lupus erythematosus cases

**DOI:** 10.1016/j.ebiom.2026.106209

**Published:** 2026-04-01

**Authors:** Maud Tusseau, Samira Khaldi-Plassart, Audrey Labalme, Anne-Laure Mathieu, Quentin Riller, Corentin Molitor, Thomas Simonet, Sebastien Viel, Christine Gaboriaud, Nicole Thielens, Lionel Heiser, Emilie Chopin, Isabelle Rouvet, Nicole Fabien, David Goncalves, Véronique Fremeaux-Bacchi, Carine El-Sissy, Nicolas Pottier, Romain Larrue, Bruno Ranchin, Audrey Laurent, Marine Fouillet-Desjonqueres, Maurine Jouret, Arsène Mekinian, Motoi Yamashita, Tomohiro Morio, Eric Hachulla, Isabelle Melki, Isabelle Kone-Paut, Claire Ballot, Heloise Reumaux, Pascal Pillet, Jerome Harambat, Christophe Richez, Olivier Richer, Yves Hatchuel, Ferielle Louillet, Jean-Christophe Lega, Isabelle Durieu, Anne Welfringer-Morin, Capucine Picard, Wassila Messadi, Françoise Sarrot-Reynauld, Damien Sanlaville, Brigitte Bader-Meunier, Thierry Walzer, Gaëtan Lesca, Frédéric Rieux-Laucat, Alexandre Belot, GENIAL/LUMUGENE Collaboratorsai, Florence Aeschlimann, Lise Allard, Laurent Besson-Leaud, Mélanie Blin, Karine Brochard-Payet, Antoine Briantais, Guylhène Bourdat, Stéphane Burtey, Alexandra Cambier, Aurélia Carbasse, Wadih Abou Chahla, Bilade Cherqaoui, Éloïse Colliou, Stéphane Decramer, Véronique Despert, Catherine Didailler, Olivier Dunard, Perrine Dusser-Benesty, Camille Faudeux, Hugues Flodrops, Etienne Ghrenassia, Olivia Gillion-Boyer, Fitsum Guebre-Egziabher, Philippe Guilpain, Vincent Guigonis, Rose-Marie Herbigneaux, Dirk Holzinger, Raju Khubchandani, Charlotte Kevorkian-Verguet, Martin Killian, Noémie Le Gouellec, Irène Lemelle, Iona Madden, Hazem Manadile, François Maurier, Ulrich Meinzer, Sandrine Morell-Dubois, Antoine Mouche, Anne-Sophie Parentelli, Alice de Pellegars Malhortie, Isabelle Pellier, Pierre Quartier dit Maire, Karine Retornaz, Linda Rossi, Caroline Rousset-Rouvière, Adrien Subervie, Soizic Tiriau, Florence Uettwiller, Diego Urbina, Jean-François Viallard, Franck Zekre

**Affiliations:** a Genetics Department, Lyon University Hospital, Lyon, France; b CIRI, International Center of Research in Infectiology, Lyon University, INSERM U1111, CNRS UMR 5308, ENS, UCBL, Lyon, France; c National Reference Center for Rheumatic, Systemic Autoimmune Diseases and Interferonopathies in Children (RAISE), Hospices Civils of Lyon, Lyon, France; d Sorbonne Université, Assistance Publique Hôpitaux de Paris, GHU Sorbonne Site Pitié-Salpêtrière, Service de Médecine Interne 2, Maladies Autoimmunes et Systémiques, Centre de Référence Maladies Auto-Immunes et Auto-inflammatoires Systémiques Rares d’Ile de France, Centre et Martinique, Centre de Référence Histiocytoses, 75651 Paris CEDEX 13, France; e Department of Bioinformatics, Hospices Civils de Lyon, France; f Hospices Civils de Lyon, Groupement Hospitalier Centre, Plateforme ARTEMIS, Lyon, France; g Institute of Structural Biology (IBS), University Grenoble Alpes, CEA, CNRS, F-38000, Grenoble, France; h Centre de Biotechnologie Cellulaire and CBC BioTec Biobank, Centre de Ressources Biologiques, Hospices Civils de Lyon, 69229, Lyon, France; i Immunology Department, Lyon-Sud Hospital, Hospices Civils de Lyon, 69149, Pierre Bénite, France; j Department of Immunology, Assistance Publique- Hôpitaux de Paris (AP-HP), Georges Pompidou European Hospital, Paris, France; k Univ. Lille, CNRS, Inserm, CHU Lille, Institut Pasteur de Lille, UMR9020-U1277 - CANTHER - Cancer Heterogeneity, Plasticity and Resistance to Therapies, Toxicology and Genetic Diseases Department, Lille, France; l Pediatric Nephrology Rheumatology Dermatology Unit, Lyon University Hospital, Bron, France; m Department of Internal Medicine, Sorbonne University, Hôpital Saint-Antoine AP-HP, Paris, France; n Laboratory for Transcriptional Regulation, RIKEN Center for Integrative Medical Sciences (IMS), Yokohama, Kanagawa, Japan; o Department of Pediatrics and Developmental Biology, Graduate School of Medical and Dental Sciences, Institute of Science Tokyo, Tokyo, Japan; p Laboratory of Immunology and Molecular Medicine, Advanced Research Initiative, Institute of Science Tokyo, Tokyo, Japan; q Department of Internal Medicine and Clinical Immunology, CHU Lille, Lille, France; r Department of General Pediatrics, Paediatric Rheumatology Unit, Armand Trousseau Hospital, Assistance Publique-Hôpitaux de Paris (AP-HP), Sorbonne Université, FHU INFLAMME, Paris, France; s Department of Paediatric Rheumatology, Hôpital de Bicêtre, APHP, Le Kremlin Bicêtre, France; t Department of Pediatrics, Jean-Minjoz Hospital, Besançon, France; u Department of Pediatric Rheumatology, Hôpital Jeanne de Flandre, CHU Lille, Lille, France; v Department of Pediatrics, Pellegrin Hôpital des Enfants, University Hospital of Bordeaux, Bordeaux, France; w Service de Rhumatologie, Centre de Référence des Maladies Auto-Immunes Systémiques Rares RESO, Hôpital Pellegrin, Réseau F-Crin CRI-IMIDIATE, & ImmunoConcEpT, CNRS UMR 5164, Université de Bordeaux, Bordeaux, France; x Department of General Pediatrics, Martinique University Hospital, Fort-de France, France; y Department of Pediatrics, Rouen University Hospital, Rouen, France; z Department of Rheumatology, Centre Hospitalier Lyon Sud, Hospices Civils de Lyon, Lyon, France; aa Department of Internal and Vascular Medicine, Hospices Civils de Lyon, Lyon, France; ab INSERM U1290, Université Claude Bernard Lyon 1, Lyon; ac Department of Dermatology, Hôpital Necker-Enfants Malades, APHP-Centre, Paris, France; ad Pediatric Hematology-Immunology and Rheumatology Department, Assistance Publique Hôpitaux de Paris (APHP), Necker University Hospital, Paris, France; ae Study Center for Primary Immunodeficiencies, (APHP), Necker-Enfants Malades Hospital, Université Paris Cité, Paris, France; af Department of Pediatrics, University Hospital Center Issaad Hassani, Beni Messous, Alger, Algeria; ag Department of Internal Medicine, University Hospital of Grenoble Alpes, Grenoble, France; ah Université Paris Cité, Immunogenetics of Pediatric Autoimmunity Laboratory, Institut Imagine, INSERM UMR1163, Paris, France

**Keywords:** Monogenic lupus, Exome sequencing, Childhood-onset SLE, Inborn errors of immunity, Autoinflammatory disorders, Auto-immunity

## Abstract

**Background:**

Systemic lupus erythematosus (SLE) is a chronic, multi-organ autoimmune disease characterised by a highly heterogeneous presentation. Specific genetic variations predispose patients to the disease, and rare monogenic forms caused by single-gene variations have been identified in a small percentage of patients, often with early disease onset. In this study, we used exome sequencing in a large cohort of patient with juvenile-onset SLE to gain insight into the genetic basis of juvenile SLE (jSLE).

**Methods:**

Patients were selected if disease onset occurred before the age of 18. We performed exome sequencing on 263 individuals across 172 distinct families. The majority of cases were solo exomes (n = 118), while others included affected duos, trios, or multiplex families (n = 18 + 5 + 1), as well as classical trios with unaffected parents (n = 30).

**Findings:**

A molecular diagnosis consistent with the clinical presentation was established in 17 patients from unrelated families (10%). Among them, we identified pathogenic or likely pathogenic variants in genes previously associated with monogenic lupus, including a novel *C1QA* variant as well as other lupus-associated genes (*COPA*, *ADAR, TLR7*, *IKZF3, RELA, PTPN11, SERPING1*). Strikingly, exome sequencing also revealed variants in immunodeficiency-associated genes (*IRAK4, USB1*), autoinflammatory disorders (*PSTPIP1*) and unexpected candidates like *ETV6*, and *MAN1B1* revealing previously unrecognised pathways in SLE development. Syndromic features and very early-onset (before the age of 5) were strongly associated with a higher diagnostic yield, reaching nearly 33% in these subgroups.

**Interpretation:**

This study expands our understanding of causes of lupus, highlighting its genetic heterogeneity. It also supports the systematic use of genetic testing in cases of juvenile lupus, especially those with very early onset or syndromic features, regardless of the clinical presentation. Given the range of unexpected molecular diagnoses identified in this study, pangenomic analysis such as exome or genome sequencing appears to be the most appropriate approach in these cases.

**Funding:**

This work was supported by: The Institut National de la Santé et de la Recherche Médicale (INSERM); Government grants managed by the Agence Nationale de la Recherche (ANR) as part of the “Investment for the Future” program: Institut Hospitalo-Universitaire Imagine (ANR-10-IAHU-01), Recherche Hospitalo-Universitaire (ANR-18-RHUS-0010); The Centre de Référence Déficits Immunitaires Héréditaires (CEREDIH); The Fondation pour la Recherche Médicale (FRM: EQU202103012670, FDM202006011291); French and European grants managed by the ANR: ANR-14-CE14-0026 (Lumugène), ANR-21-CE17-0064 (SOCSIMMUNITY); The National Reference Center for Rheumatic, Autoimmune and Systemic Diseases in Children (RAISE).

**Research in context**

**Evidence before this study**

Prior studies using targeted gene panels in unselected juvenile-onset SLE cohorts identified monogenic causes in approximately 7% of cases, implicating pathways such as complement deficiency, nucleic acid sensing and adaptive immunity.

**Added value of this study**

This cohort of 172 jSLE and 91 relatives represents the largest exome-based investigation of unselected jSLE, revealing a molecular diagnosis in 10% of cases. Beyond confirming known pathways, we identified novel variants in *SOCS1* and *PTPN2* (JAK-STAT regulation) and *DOCK11*, *ETV6* (cytoskeletal dynamics), expanding jSLE's genetic architecture to include checkpoints dysregulation and cellular motility defects.

**Implications of all the available evidence**

Our findings suggest that jSLE may represent a convergence of genetically distinct disorders with shared autoimmune features. The 10% diagnostic yield supports integrating exome sequencing into routine jSLE evaluations as this information enables tailored therapies, such as JAK inhibitors for *SOCS1* or *PTPN2* haploinsufficiency.

## Introduction

Systemic lupus erythematosus (SLE) (OMIM#152700), or lupus, is a chronic autoimmune disorder that can affect various organs such as the skin, joints, kidneys, lungs, and brain. SLE is characterised by the production of autoantibodies, particularly antinuclear antibodies (ANA), which serve as key immunological biomarkers for the disease. Its progression is marked by recurrent flares followed by remission, with severity showing considerable inter-individual variability among patients. The prevalence of lupus is higher in women than in men, with an adult female-to-male ratio of approximately 9:1. Childhood-onset lupus, also known as juvenile systemic lupus erythematosus (jSLE), refers to disease onset before the age of 18 years. The specificities of jSLE include a more severe disease with a higher frequency of kidney and neurological involvement and a female-to-male ratio lower than that in adults.^1^^,^^2^ jSLE is also associated with increased long-term morbidity and mortality.^3^^,^^4^ A deeper understanding of its underlying causes and therapeutic targets remains an important unmet need.

The development of SLE is driven by a complex interplay of genetic, environmental, and hormonal factors that influence both age of onset and disease severity. The causal role of genetic factors in SLE is supported by genome-wide association studies (GWAS) that have identified many susceptibility *loci*, most of which are linked to immunity-related genes.^5^, ^6^, ^7^, ^8^ In addition, monogenic forms of SLE have been described, starting with complement deficiencies identified in the 1970s, and many more since then, particularly following the advent of next-generation sequencing.^9^, ^10^, ^11^ Monogenic SLE is caused by single-gene defects and is frequently associated with early-onset or familial transmission. SLE-causing genes have been mapped to multiple pathways, including the complement cascade, the regulation of type I IFN production and the response to this cytokine,^12^, ^13^, ^14^ B-cell physiology, JAK-STAT signalling or TLR pathways.^15^, ^16^, ^17^, ^18^, ^19^, ^20^ The great diversity of these monogenic forms highlights a broad range of underlying disease conditions.

In 2020, our team reported the results of a genetic panel analysis of 117 jSLE cases and identified monogenic forms in approximately 7% of these paediatric cases.^21^ Here, we performed exome sequencing on a cohort of 172 jSLE cases as part of an ongoing effort to improve the molecular diagnosis rate in patients with suspected monogenic lupus.

## Methods

### Ethics

Patients were recruited in the French national cohort GENIAL (GENetic & Immunologic Anomalies in systemic Lupus erythematosus) from hospitals across France (Assistance Publique—Hôpitaux de Paris (AP-HP), Hospices Civils de Lyon (HCL), CHU Lille, CHU Toulouse, CHU Bordeaux, CHU La Réunion, CHU Rennes, Assistance Publique—Hôpitaux de Marseille (AP-HM), CH Côte Basque, CHU Grenoble Alpes, CHRU Brest, CHRU Nancy, CHU Rouen, CHRU Besançon, CHR Metz-Thionville, CHU Montpellier, CHU Angers, CHRU Tours, CHU Nantes, CH Annecy-Genevois/Chambéry, CHU Limoges, CHU Martinique, CH Avignon/Marseille, Centre Hospitalier de Saint-Omer) and Algeria (University Hospital Center Issaad Hassani, Algeria). Written informed consent was obtained from the patients’ parents or from the patients themselves for patients aged over 18 years. Patients provided written consent for genetic analysis and collection of clinical and biological data from medical records (age of onset, consanguinity, familial history of auto-immune diseases, type of organ involvement, and treatment at the time of inclusion). Sex was determined by the physician from clinical records and recorded as female or male. No gender identity data were collected, and sex-stratified analyses were not performed. Ethical approval was obtained from the French Ethics Committee of CPP-SUD-Est III (2013). This study was registered under the EudraCT number (2012-A01449-34) and NCT01992666. Informed consent for publication of patient images was obtained from all patients and their parents.

### Patients’ selection

Probands were included in the biological collection if they presented with paediatric-onset systemic lupus (according to ACR or SLICC criteria), syndromic forms of lupus (defined as cases presenting with non-immune-mediated symptoms in addition to lupus features, such as neurodevelopmental delay, growth abnormalities, or congenital anomalies), or a family history of autoimmune diseases. Family history was documented at two levels: (1) broad immune dysregulation, including rheumatic and non-rheumatic autoimmune diseases in first and higher-degree relatives; and (2) specific history of lupus in first-degree relatives. Consanguinity was recorded based on patient self-report; no genetic verification was performed.

Patients were eligible for inclusion in the genetic study if disease onset occurred before 18 years of age, without sex-based exclusion. Retrospective data from medical records drove recruitment, focussing on clinical/genetic factors over sex stratification. Exclusion criteria included incomplete clinical data, an uncertain diagnosis of SLE, or a previously established genetic diagnosis obtained through external analysis.

### Exome sequencing

**Fig. 1:**
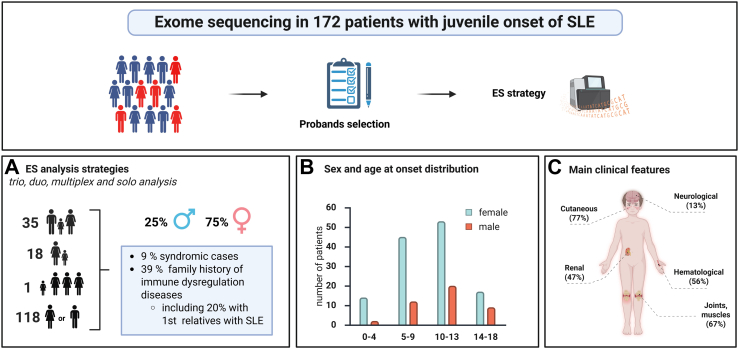
**Study design.** 172 probands were selected based on juvenile-onset lupus. **A:** Exome sequencing (ES) analysis strategies. **B:** Sex and age distribution in the cohort. **C:** Main clinical features based on the type of organ involvement.

Exome sequencing was performed on 172 probands and their available relatives (affected or unaffected), resulting in a cohort of 263 individuals, including 202 patients with lupus. Most probands underwent solo exome sequencing (n = 118), while 35 were sequenced as part of trios, duos (n = 18), or multiplex families (n = 1) (Fig. 1A). DNA samples were sequenced using Illumina technology, using different capture kits: KAPA HyperExome or SeqCap EZ MedExome (Roche, 09062599001, 06392695001), SureSelectXT HS Target Enrichment System (Agilent, 5190-6209, 5190-8864) or Exome plus (Twist, 109326) on different sequencers (NovaSeq 6000, NextSeq 500, and HiSeq 2500). Sequences were aligned with the hg19 reference human genome using Burrows-Wheeler Alignment (BWA) and variant calling was performed using the Genome Analysis Toolkit (GATK). All variants were annotated using a software system developed by the Paris Descartes University bioinformatics platform or the in-house bioinformatics pipeline of Lyon University Hospital.

### Identification of disease-causing variants

We first considered the following genes in our genetic analysis: (1) Genes already identified in monogenic SLE; (2) Genes responsible for inborn errors of immunity (IEI) as updated in 2022^22^ and; (3) Putative lupus-causing genes, selected from GWAS and mouse models (Lists described in the Supplementary Method). We then explored *de novo* variants identified in trios and investigated loss-of-function variants across the entire dataset to identify potential new lupus-causing genes. Variants previously classified as pathogenic or likely pathogenic in the ClinVar database were systematically reviewed in relation to the patient's clinical phenotype. For List 1, variants were examined if they had a minor allele frequency (MAF) <1% in the gnomAD v2 database and fewer than 10 occurrences in the internal variant database. For Lists 2 and 3, more stringent thresholds were applied: MAF <0.1% for dominant inheritance models or <1% for recessive models in public databases, internal occurrence <5, and a CADD score >20 for missense variants. In the non-targeted analysis, variants were selected if they had fewer than 5 alleles in gnomAD (for dominant inheritance), and a CADD score >20 was required for missense variants.

The classification of variants was based on clinical practice recommendations of the American College of Medical Genetics (ACMG).^23^ Familial segregation for patients tested by solo exome sequencing was assessed by targeted Sanger sequencing of the identified variant.

### RNA assay for splicing analysis

RT-PCR was performed on 250 ng of total RNA extracted from the patient's whole blood collected in PaxGene tube and from a control sample, using the Transcriptor High Fidelity cDNA Synthesis Kit (Roche, 4368814), following the manufacturer's protocol. cDNA amplification was subsequently carried out using GoTaq G2 DNA Polymerase (Promega, M7841).

### IKZF3 expression

The plasmid pTwist CMV, with either wild-type or mutated *IKZF3* (NM_001257414) cDNA were synthesised by Twist Biosciences (San Francisco, CA, USA). An HA-tag was added to the N-terminal region of the cDNA sequence. HEK293T (RRID:CVCL 0063) cells were seeded in a 6-well plate (0.5 × 10^6^ cells per well) and transfected using the JET-PEI reagent (JetPEI Polyplus Transfection Kit, Sartorius, 101000020) following the manufacturer's instructions. Cell lines were routinely tested for mycoplasma contamination and were negative. Cells were lysed in RIPA (25 mM Tris, HCl, pH 7.4, 150 mM NaCl, 1% NP-40, 1% sodium deoxycholate and 0.1% SDS containing protease and phosphatase inhibitors (Sigma–Aldrich)) and protein levels were measured using a μBCA Protein Assay Kit (Thermo Fisher Scientific, 23235). Protein expression was evaluated using the Jess™ Simple Western system (ProteinSimple, San Jose, CA, USA). Briefly, cell lysates were diluted and mixed with sample buffer and Fluorescent 5X Master Mix and then heated at 95 °C for 5 min. Proteins were separated in capillaries using the Jess 12-230-kDa separation module. Detection was performed using an anti-IKZF3 (1:100, Novus-Bio, Ref NBP2-16938) and anti-HA tag antibodies (1:40, Cell Signalling Technology, ref: CST#3724), followed by HRPO-conjugated secondary antibodies (ProteinSimple, San Jose, CA, USA, Ref: DM-001). The chemiluminescence signal in the capillaries was digitally analysed using the Compass Simple Western software (version 4.1.0, ProteinSimple). The software automatically calculated the signal intensity, area, and signal-to-noise ratio (SNR). The results are displayed as electropherograms showing peaks of signal intensity and lane views of the detected chemiluminescence.

### Type I interferon signature

The type I interferon signature, reflecting IFN pathway activation, was measured using NanoString or RT-qPCR as previously described.^24^ The score is based on the expression levels of a set of interferon-stimulated genes in the blood cells. The expression of these genes is compared to a reference group.

### Transferrin isoelectric focussing

Serum transferrin isoforms were separated by isoelectric focussing on agarose gel using PhastSystem (Amersham Biosciences) as previously described.^25^

### Statistical analysis and figures

All statistical analyses were performed using GraphPad Prism (version 10). For the comparison of mean ages between cohorts, we used Welch's t-test, which does not assume equal variances. The significance threshold was set at 0.05. For the comparison of inclusion criteria and the percentage of genetic forms, Fisher's exact tests were performed with a significance threshold of 0.01. Figures were created with BioRender software (https://biorender.com).

### Role of the funders

The funders had no role in study design, data collection, analysis, interpretation, or writing of the report.

## Results

### Cohort of patients

The cohort comprised 172 probands with jSLE and additional relatives diagnosed with SLE, totalling 202 SLE patients. Thirty-six percent of the probands (63/172) had a relative with a family history of immune dysregulation disease, including 18% (32/172) with a first-degree relative diagnosed with SLE. Consanguinity was observed in 12% of probands. The female-to-male ratio was 4:1 and the mean age at disease onset among probands was 9.7 ± 3.6 years (Fig. 1B).

We observed a frequency of organ involvement comparable to that seen in paediatric cases with renal involvement in 47% of cases, haematological abnormalities in 56%, and neurological manifestations in 13% (Fig. 1C). Additionally, 15 patients (9%) presented with a syndromic phenotype characterised by features unrelated to SLE, such as neurodevelopmental disorders, seizures, congenital cardiopathy, or growth delay (see Supplementary Table S1 for details).

**Fig. 2:**
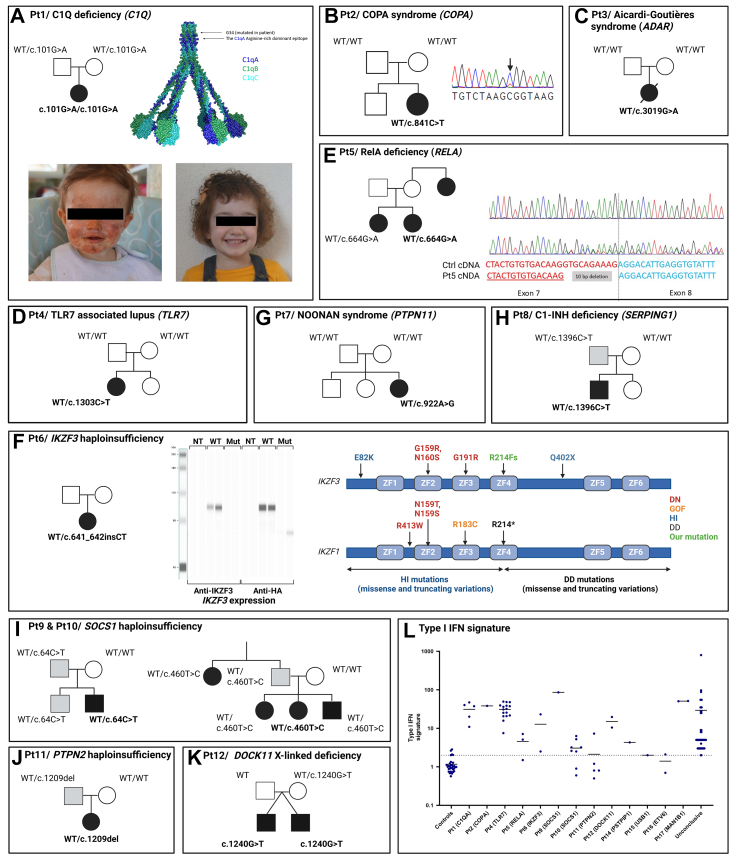
**Pathogenic or likely pathogenic gene variations in 11 SLE patients. A–K:** Pedigrees of families with a molecular diagnosis of lupus (Black: affected patients; grey: unaffected variation carriers; WT: wild-type; proband's genotype shown in bold). Patients 9–12 were previously reported.^16^^,^^26^, ^27^, ^28^**A, top right:** Position of the G34 mutation in the overall C1q model. This residue lies in the bundle region where the six collagens assemble together and precedes the arginine-rich dominant epitope in C1qA. **A, lower panel:** Cutaneous involvement **(left)** and remission after haematopoietic stem cell transplantation **(right)**. **B:** Sanger trace of the *COPA* variation relative to a somatic variation. **D:** Sanger trace of the patient's cDNA compared and control, showing the splicing impact of the *RELA* variant, with a 10 bp deletion in exon 7 (NT: not transfected). **F, middle:***IKZF3* expression in HEK293T cells transfected with WT or mutated constructs and revealed with anti-HA (left) or anti-IKZF3 (right) antibodies. **F, right:** Linear representation of *IKZF3* and *IKZF1* functional domains and previously published variations (DN: dominant negative; GOF: gain-of-function; HI: haploinsufficiency; DD: dimerisation defective). **L:** Type I IFN signature of patients and controls. Each point represents a measurement at different time points. The dotted line indicates the positive cut-off (2.3).

A molecular diagnosis consistent with the clinical presentation was established in 17 patients from unrelated families, representing 10% of the 172 individuals with jSLE (Table 1). Among the cases described in this manuscript, patients 2, 4 and 9–12 have been previously reported, while the remaining cases represent novel, unreported patients.^16^^,^^26^, ^27^, ^28^, ^29^ In the following section, we report the results of genetic and functional testing along with the observed phenotype for each of these patients. Type I interferon signatures, when available, are represented in Fig. 2L.

**Table 1:** Clinical and biological data in 17 patients with identified Pathogenic or probably pathogenic variants.

Patient	Gender	Clinical presentation	Outcome	Biological findings	Age at lupus onset	Family history of auto-immune disease	Variants of interest^a^	Type of analysis Variants transmission	ACMG Class	Associated disease	Publication
Pt1	F	Cutaneous lupus	Remission after HSCT	CH50 ↓, C3 N, C4 N Positive ANA, negative anti ds-DNA Positive type I IFN signature (20.1; 37.6; 47.1; 10.9; NR < 2.3)	9 months	No	***C1QA*(NM_015991.4):c.101G>A p.(Gly34Glu), homozygous**	Trio Maternal and paternal	Likely pathogenic	C1q deficieny	
Pt2	F	ANCA-associated vasculitis with lupus glomerulonephritis	Remission without immunosuppressive treatment	C3 N, C4 ↓ Positive p-ANCA, ANA and anti ds-DNA Positive type I IFN signature (38.3)	2 years	No	***COPA*(NM_004371.4):c.841C>T p.(Arg281Trp)**	Trio *De novo*	Likely pathogenic	COPA syndrome	Tusseau et al.
Pt3	F	Syndromic lupus (encephalopathy, chilblains, cardiac involvment)	Death from acute cardiac injury	CH50 ↓, C3 N, C4 N Positive ANA, negative anti ds-DNA Positive Coombs and antiphospholipid antibodies IFN-α (SIMOA): 433.8 fg/mL	12 years	No	***ADAR*(NM_001111.5):c.3019G>A p.(Gly1007Arg)**	Trio *De novo*	Pathogenic	Aicardi-Goutieres syndrome	
Pt4	F	Syndromic lupus with psychomotor delay, lupus nephritis and recurrent infections	Disease controlled by hydroxychloroquine, enalapril, mycophenolate mofetil and belimumab	CH50 ↓, C3 ↓, C4 ↓ Positive ANA, negative anti ds-DNA, positive anti-C1q antibodies, type 3 cryoglobulinemia Positive type I IFN signature (9 values above 30)	2 years	No	***TLR7*(NM_016562.4):c.1303C>T p.(Pro435Ser)**	Solo *De novo*	Likely pathogenic	Systemic lupus erythematosus	Tusseau et al.
Pt5	F	Lupus with initial articular and haematological involvement; progression to cutaneous, pulmonary, serosal, and renal manifestations; basal ganglia calcifications.	Partial response to hydroxychloroquine, corticosteroids, rituximab and MMF, then introduction of anifrolumab	C3 slightly ↓, C4 N Positive ANA, positive anti ds-DNA (31, NR <10) and anti Sm Positive type I IFN signature (7.1–5.1), then negative (1.5)	15 years	Sister with unclassified inflammatory disease, aunt with lupus	***RELA*(NM_021975.4):c.664G>A p.Val219ArgfsTer94**	Solo Unknown	Likely pathogenic	RelA haploinsufficiency	
Pt6	F	Lupus with cutaneous, renal, articular and haematological involvment	Disease controlled by corticosteroids	CH50 ↓, C3 ↓, C4 ↓ Positive ANA Positive and slightly positive type I IFN signature (2.5; 23.1)	10 years	No	***IKZF3*(NM_012481.5):c.641_642insCT (p.Arg214SerfsTer2)**	Solo Unknown	Likely pathogenic	AIOLOS haploinsufficiency	
Pt7	F	Lupus with renal, haematological, pulmonar involvment; short stature (−3.7. SD at lupus diagnosis)	Disease controlled by cyclophosphamide corticosteroids, hydroxychloroquine and MMF	Positive ANA, anti ds-DNA, anti-SSA and anti-SM	14 years	No	***PTPN11*(NM_002834.5):c.922A>G p.(Asn308Asp)**	Trio *De novo*	Pathogenic	Noonan syndrome	
Pt8	M	Lupus with cutaneous, renal and joint involvment	Treatment with corticosteroids, hydroxychloroquine and MMF; disease controlled with hydroxychloroquine and MMF	C3 N, C4 ↓ Positive ANA, anti ds-DNA, anti-SSA and SSB	14 years	Paternal grandfather with dermatomyositis	***SERPING1*(NM_000062.3):c.1396C>T p.(Arg466Cys)**	Trio Paternal	Pathogenic	C1 inhibitor deficiency/Hereditary angioedema	
Pt9	M	Lupus with renal involvment and growth failure due to growth hormone deficiency	Remission	Positive ANA and anti ds-DNA Positive type I IFN signature (86)	16 years	No	***SOCS1*(NM_003745.2):c.64C>T p.(Arg22Trp)**	Solo Paternal	Likely pathogenic	SOCS1 haploinsufficiency	Hadjadj et al., (patient D1)
Pt10	F	Lupus with renal, articular and cutaneous involvment	Treatment with baricitinib and then benlysta	C3 ↓, C4 ↓ Positive ANA and anti ds-DNA Fluctuating type I IFN signature (3.2; 0.6; 6.3; 3.2; 0.9; 2.6; 2.5; 5.2)	9 years	Sister with ITP, brother with psoriasis	***SOCS1(NM_003745.2):c.460T>C p.(Tyr154His)***	Solo Paternal	Likely pathogenic	SOCS1 haploinsufficiency	Hadjadj et al., (patient E1)
Pt11	F	Lupus with hepatic, renal and haematological involvment	Disease controlled by corticosteroids, hydroxychloroquine and azathioprine	CH50 ↓, C3 ↓, C4 ↓ Positive ANA, negative anti ds-DNA Positive anti B2Gp1, anti C1q Positive coombs Positive type I IFN signature once (7.3) and negative (0.8; 0.5; 0.8; 1.2)	5 years	No	***PTPN2*(NM_002828.4):c.1209delp.(Phe403LeufsTer25)**	Solo Paternal	Likely pathogenic	PTPN2 haploinsufficiency	Jeanpierre et al., (patient A2)
Pt12	M	Mixed connective tissue disease and bullous systemic lupus erythematosus	Treatment with corticosteroids, hydroxychloroquine and dapsone	CH50, C3, and C4 N Positive ANA Positive type I IFN signature (19.7)	9 years	Dizygotic twin brother diagnosed with a mixed connective tissue disease at 14 y.o.	***DOCK11*(NM_144658.4):c.1240G>Tp.(Asp414Tyr), hemizygous**	Duo Maternal (X-linked)	Likely pathogenic	DOCK11 deficiency	Boussard et al., (patient D2)
Pt13	F	Lupus with cutaneous involvment, history of infections	Partial disease response with hydroxychloroquine	Positive ANA, negative anti ds-DNA Positive type I IFN signature	1 year	Sister with cutaneous lupus	***IRAK4*(NM_016123.4):c.877C>T p.(Gln293Ter), *IRAK4*(NM_016123.4):c.652del p.(Met218TrpfsTer9)**	Duo Maternal Paternal	Pathogenic Pathogenic	IRAK4 deficiency	
Pt14	F	Articular involvement, rosacea-like cutaneous lesions, macrophage activation syndrome	Disease controlled by IL-1 inhibitors	Positive ANA, negative anti ds-DNA Profound neutropenia Slightly positive type I IFN signature (4.4) Elevated zincemia: 3548 μg/L (NR:553-1046)	4 years	No	***PSTPIP1*(NM_003978.5):c.748G>A p.(Glu250Lys)**	Unknown	Pathogenic	PAMI syndrome	
Pt15	F	Skin involvement, recurrent infections and hepatosplenomegaly	Initial treatment with corticosteroids	Anaemia and neutropenia Positive ANA (anti SSA) Slightly positive type I IFN signature (3)	1 year	No	***USB1*(NM_024598.4):c.502del p.(Arg168GlyfsTer97), homozygous**	Maternal and paternal	Pathogenic	Poikiloderma with neutropenia	
Pt16	F	Immune thrombocytopaenia and mild cutanous involvment (photosensibilisation)	Increase in platelet count with hydroxychloroquine and IVIg (100–150,000 platelets/μL)	CH50 N, C3 N, C4 slightly ↓ Positive ANA, negative anti ds-DNA Lowest platelet counts: 8, 000/μL Negative type I IFN signature (0.7; 2.1)	10 years	No	***ETV6*(NM_001987.5):c.1123G>A p.(Gly375Arg)**	Trio *De novo*	Likely pathogenic	Dominant thromobocytopenia	
Pt17	F	Global developmental delay, tall stature (+4 SD), obesity (+5 SD), precocious puberty, cutaneous lupus with malar rash and chilblains	Remission of cutaneous lupus with hydroxychloroquine	CH50 N, C3 N, C4 N Positive ANA and anti ds-DNA Positive type I IFN signature (51.3; 50.9)	7 years	No	***MAN1B1*(NM_016219.5):c.1833_1834del p.(Asp613ProfsTer40), homozygous**	Trio Maternal and paternal	Pathogenic	Rafiq syndrome	

ANA: Antinuclear Antibodies; anti-dsDNA: Anti–Double-Stranded DNA; CH50: Haemolytic Complement Activity; HSCT: Haematopoietic Stem Cell Transplantation; IFN: Interferon; MMF: Mycophenolate Mofetil; p-ANCA: Perinuclear Anti–Neutrophil Cytoplasmic Antibodies; NR: Normal Range; SD: Standard Deviation.

a Unless specified, variants are heterozygous.

### Pathogenic or likely-pathogenic variations in genes previously associated with SLE

The first case is a confirmed C1q deficiency in a girl with very early onset SLE, which began at 9 months of age, with erythematous-squamous facial lesions, oral ulcerations, and growth failure (Fig. 2A). As observed in other C1q-deficient patients, she presented with positive ANA with anti-SSA, anti-Sm and anti-RNP but negative anti-double-stranded DNA antibodies (anti-dsDNA). She carries a homozygous missense variation in *C1QA* (c.101G>A, p.(Gly34Glu)), not yet described in the literature, *C1QA* encoding one of the three chains composing the C1q protein. This variant has only been reported once in heterozygous state in the gnomAD v4.1.0 database and is predicted to be damaging according to multiple *in silico* scores (CADD Phred: 25.20; AlphaMissense: 0.748, classified as Likely Pathogenic; and REVEL: 0.622). Glycine substitution is structurally detrimental to the correct organisation of collagen-type chains and is predicted to affect the assembly of the three chains of the C1q molecule. Notably, different amino acid change but affecting the same amino acid residue is known to be pathogenic in the *C1QC* gene, encoding another of the three C1q chains.^30^ Moreover, quantitative complement analysis revealed persistently low C1q levels despite normal C3 and C4 levels, along with consistently reduced CH50 levels (14–15 μ/mL, normal range 41–95 μ/mL; 0% haemolytic activity, normal range 70–130%). Functional complement studies with reconstitution of the missing protein confirmed a C1q deficiency.

We also identified variants in two genes associated with type I interferonopathies; the first one is a *de novo COPA* variant (c.841C>T, p.(Arg281Trp)) identified in Pt2. This variant was previously identified in a boy with deforming arthritis whose father developed progressive lung disease, and in another boy with follicular bronchiolitis, positive ANA and anti-neutrophil cytoplasmic antibodies (ANCA).^31^^,^^32^ This variant is located in a mutational hotspot region within exons 8–9 of the *COPA* gene and considered likely pathogenic. The variation was found to be somatic as indicated by the Sanger trace and an allele frequency of 30% in the exome dataset (Fig. 2B) and published elsewhere with another case.^29^ Patient 2 was a girl diagnosed with ANCA vasculitis and glomerulopathy at the age of 2 years. Then, she presented with positive ANA, Coombs and C4 diminution leading to the diagnosis of SLE. The second case was a pathogenic variant in the Aicardi-Goutières Syndrome (AGS) gene *ADAR* (c.3019G>A, p.(Gly1007Arg)) identified in a girl (Pt3) with a severe neurodevelopmental disorder from birth, initially attributed to a putative congenital infection (Fig. 2C).^13^ At the age of 12 years, she developed SLE with cutaneous and cardiac involvement and later experienced a fatal cardiac arrest during an inflammatory flare. This genotype provided a definitive diagnosis for both encephalopathy and SLE.

Then, we identified a novel *TLR7* variant (c.1303C>T, p.(Pro435Ser)) in a 6-year-old girl with syndromic lupus (Pt4) characterised by significant psychomotor developmental delay and pyramidal syndrome (Fig. 2D). The variant, initially considered as a variant of uncertain significance was found to be *de novo* through trio genome sequencing. This variant was absent from the gnomAD database (v4.1.0) and showed discordant predictions according to multiple *in silico* scores (CADD Phred: 20.10; AlphaMissense: 0.412; and REVEL: 0.128). Given its rarity and *de novo* origin, it fulfilled the criteria for reclassification as likely pathogenic. At the age of two, she presented with lupus nephritis and autoimmune cytopenia, requiring multiple lines of B-cell–targeted therapy. She also developed an immunoglobulin deficiency and, despite supplementation, experienced multiple infections. The full clinical details of this case and functional characterisation of the variant have been reported.^28^

We also identified a heterozygous variant in *RELA*, which encodes the p65 subunit of the NF-κB pathway. The variant (c.664G>A) absent from the gnomAD V4.1.0 database, is located at the end of exon 8 and is predicted to affect splicing. The patient (Pt5) presented with SLE with multi-organ involvement, requiring multiple lines of therapy. Pathogenic heterozygous variants in *RELA* causing haploinsufficiency have previously been associated with autoinflammatory phenotypes resembling Behçet's disease.^33^ Subsequently, dominant-negative variants have been reported in patients with a potentially more severe clinical presentation.^34^ We performed RNA analysis and confirmed that the variant resulted in loss of the canonical donor splice site and the creation of a cryptic splice site, leading to the deletion of 7 base pairs from exon 7 and the introduction of a premature stop codon (r.655_664del, p.(Val219ArgfsTer94)) (Fig. 2E). This variant was also present in the patient's sister, who presented with an unspecified autoinflammatory disease, characterised by anaemia, arthralgia and oral and genital ulcers.

An *IKZF3* truncating variation absent from the gnomAD v4.1.0 database was detected in Pt6, a 10 year-old girl with SLE. *IKZF3* encodes the transcriptional factor AIOLOS, a member of the IKAROS family which was associated in 2021 with a B-cell immunodeficiency and susceptibility to EBV infection.^35^ More recently, the spectrum of diseases associated with *IKZF3* pathogenic variants has expanded to include autoimmune conditions such as Hashimoto's thyroiditis, immune thrombocytopaenia, SLE and autoimmune hepatitis, observed in 5 out of 9 patients reported.^36^ Variants associated with autoimmunity were found to result in haploinsufficiency. This mirrors the clinical observations associated with variations in *IKZF1*, which encodes IKAROS, where haploinsufficiency or dimerisation defects are associated with the development of autoimmunity rather than immune deficiency.^21^^,^^37^ Pt6 carried a two-nucleotide insertion in *IKZF3* that resulted in a frameshift. We cloned the variant and expressed it in HEK293T cells. We showed that the *IKZF3* variant led to highly reduced protein expression compared to WT IKZF3, as measured by capillary Western blot, regardless of the antibody used to detect it (anti-AIOLOS or anti-HA tag) (Fig. 2F).

A *de novo* pathogenic variant in *PTPN11* (c.922A>G, p.(Asn308Asp)) was identified in Pt7 (Fig. 2G). *PTPN11* variants are responsible for approximately 50% of Noonan syndrome cases, this syndrome being part of a group of disorders known as RASopathies. RASopathies are characterised by pathogenic gene variants in components or regulators of the RAS/MAPK signalling pathway, leading to increased activation of this pathway.^38^^,^^39^ RASopathies are frequently associated with auto-immune diseases and SLE.^40^ Short stature was the only clinical feature suggesting Noonan syndrome in the patient, even after in-depth retro-phenotyping.

In a male patient (Pt8), we identified a pathogenic variant in *SERPING1*, the gene encoding C1-inhibitor. This variant has been previously confirmed as disease-causing in hereditary angioedema (HAE) and was inherited from his asymptomatic father.^41^ The patient has SLE with cutaneous, renal, and joint involvement, and no clinical signs of HAE. HAE can be associated with incomplete penetrance, and its co-occurrence with autoimmune diseases has been previously documented, with lupus being the most frequently reported, with prevalence estimates ranging from 2% to 12%.^42^^,^^43^ Mechanistically, C1-inhibitor deficiency may contribute to SLE pathogenesis through dysregulation of the classical complement pathway, impairing immune complex clearance and fostering autoimmunity (Fig. 2H).

Finally, variants in *SOCS1*, *PTPN2,* and *DOCK11* were identified in Pts 9–12 (Fig. 2I–K). We previously reported these cases, along with a detailed analysis of the functional impact of the variations.^16^^,^^26^^,^^27^

### Variations in genes associated with primary immune dysregulation and deficiency (PIDD)

**Fig. 3:**
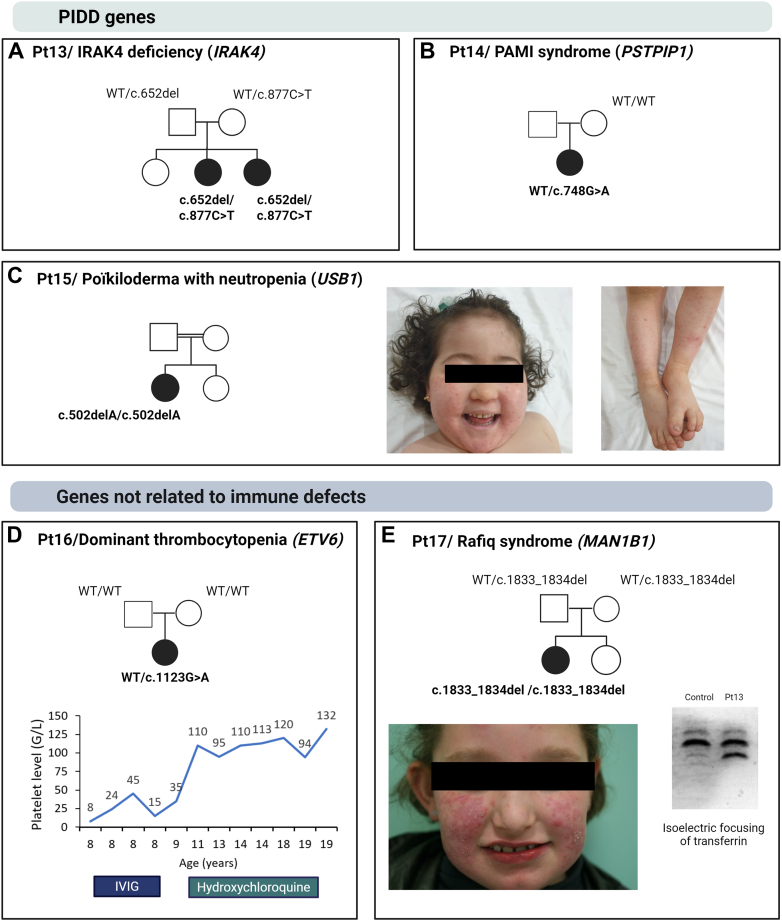
**Unexpected molecular diagnosis in SLE. A–E:** Pedigrees of families with a molecular diagnosis not related to lupus (Black: affected patients; WT: wild-type). **C:** Cutaneous involvement of Pt15. **D:** Platelet levels in patient with *ETV6* related dominant thrombocytopaenia. **E, lower left:** Cutaneous lupus in the proband. **E, lower right:** Distribution of serum transferrin glycoforms after transferrin isoelectric focussing of the patient compared to that of the control. Patient's Trf profile was abnormal, with in particular elevated 3-sialo-Trf compared with control.

For Pts 13–15, we identified disease-causing variants in two genes responsible for PIDD, that was not previously associated with SLE (Fig. 3A–C). Pt13 carried two different truncating variants in *IRAK4* gene, consistent with a diagnosis of IRAK4 deficiency (Fig. 3A). Both variants have been described as pathogenic in the literature or ClinVar in the context of IRAK4-associated immunodeficiency.^44^ Both Pt13 and her sister, who shared the same *IRAK4* genotype, presented with a history of infections and cutaneous lupus since the age of one year. IRAK4 deficient patients are more susceptible to infections, owing to the crucial role of the protein in different innate immunity signalling pathways.^45^ The association with SLE was unexpected, since IRAK4-deficient mice show less severe lupus manifestations than WT mice, and because no association between IRAK4 deficiency and a lupus phenotype was reported to date.^46^ Recently, *IRAK4* was also associated with an autoinflammatory syndrome called the NASA syndrome, secondary to the combination of a loss-of-function and a missense variation.^47^ Increase in TLR signalling is strongly implicated in SLE pathogenesis, as shown by the presence of *TLR7* gain-of-function or *UNC93B1* gain-of-function variants that enhance the TLR7/8 activity in patients. *IRAK4* pathogenic variations may further potentiate TLR7/8 hyperactivation by amplifying downstream NF-kB and interferon signalling cascades, which are initiated through UNC93B1-mediated trafficking of nucleic acid-sensing TLRs to endosomes, thus creating a synergistic loop of aberrant innate immune activation linked to autoimmunity. Further functional studies are required to elucidate the precise impact of IRAK4 defect on SLE pathogenesis.

A recurrent variant in *PSTPIP1* (c.748G>A p.(Glu250Lys)) led to the identification of an autoinflammatory disorder known as PAMI syndrome in Pt14, a girl with atypical SLE (Fig. 3B). PAMI syndrome has already been described in one patient with SLE^48^; however, it is classically associated with skin inflammation, cytopenia, growth retardation, and hepatosplenomegaly.^49^ Biologically, most patients present with hyperzincaemia and hypercalprotectinemia.^49^ The clinical and biological presentation of our patient was consistent with this syndrome, with an increase of zincaemia (3548 μg/L; NR:553-1046) and the unusual presence of ANA.

Finally, we report a novel homozygous *USB1* truncating variant, absent from gnomAD v4.1.0. This variant was identified in a girl presenting with cutaneous involvement and recurrent infections, in whom a diagnosis of SLE was initially considered due to positive ANA (Pt15, Fig. 3C). Biallelic loss-of-function variants in *USB1* cause poikiloderma with neutropenia (PN), an ultra-rare autosomal recessive disorder characterised by early-onset skin involvement, typically beginning as a papular erythematous rash with photosensitivity, along with chronic neutropenia and recurrent infections. While immune deficiency is a hallmark of the disease, autoimmune manifestations are exceedingly rare, and to our knowledge, no association with SLE has been previously reported.^50^^,^^51^

### Unexpected molecular diagnosis in lupus

Our genetic analysis also led to unexpected diagnoses in two additional patients with SLE (Fig. 3D and E). First, a *de novo*, likely pathogenic variant in *ETV6* led to a diagnosis of dominant thrombocytopaenia in Pt16. This variant has only been reported once in heterozygous state in the gnomAD v4.1.0 database and predicted to be deleterious according to *in silico* scores (CADD Phred: 32; AlphaMissense: 0.998; and REVEL: 0.514). This patient had a history of severe thrombocytopaenia and skin involvement. The thrombocytopaenia was considered immune-related because it was partially responsive to hydroxychloroquine, resulting in moderate thrombocytopaenia (Fig. 3D).

The second diagnosis unrelated to SLE was a metabolic disorder in Pt17, specifically a congenital disorder of glycosylation (CDG) called Rafiq syndrome, which is caused by a homozygous variation in *MAN1B1.* The deletion c.1833_1834del p.(Asp613ProfsTer40) was already described as disease causing.^52^ Isoelectric focussing of transferrin in the patient was consistent with this diagnosis (Fig. 3E). The patient's genotype explains the major features of her phenotype, which is characterised by a neurodevelopmental disorder associated with obesity, tall stature, and early puberty.

### Other variants of interest

Other variants of interest were identified in seven families (4%) but further evidence is required to include them as pathogenic or likely pathogenic variants. These include variants of unknown significance in *BANK1, STAT2, STAT5B, OTUD1, TNIP1,* and *COPA* (Supplementary Tables 2).

### Diagnostic yield according to inclusion criteria

In this study, patients were selected based on age at SLE onset. The clinical presentation in our cohort was similar to that described for paediatric SLE, with a high rate of organ involvement. Patients with a molecular diagnosis had an earlier disease onset compared to those without (7.70 vs. 9.95 years), although this difference did not reach statistical significance (p = 0.101, Welch's t-test).

Clinical and biological presentations, as well as family history, did not appear to differ between patients with a molecular diagnosis and those with inconclusive exome results (Table 2). However, we did not assess disease severity, which may be greater in genetically determined forms.

**Table 2:** Differences in clinical manifestations and inclusion criteria between patients with a molecular diagnosis and those with inconclusive exome results.

	Overall cohort	Positive exome	Inconclusive exome	Statistical difference between positive and inconclusive
Clinical manifestations				
Mucocutaneous	76.6%	94.1%	74.7%	ns
Musculoskeletal	66.7%	58.8%	67.6%	ns
Haematologic	56.1%	47.1%	57.1%	ns
Renal	46.6%	52.9%	45.9%	ns
Neurological	13.4%	11.8%	13.5%	ns
Inclusion criteria				
VEO-SLE	8.7%	29.4%	6.5%	∗∗ (0.0084)
Syndromic	8.7%	35.3%	5.8%	∗∗ (0.0011)
Family history	38.9%	29.4%	40.0%	ns

VEO (Very Early Onset) is defined as disease onset before 5 years of age. NS: non-significant (p ≥ 0.05).

∗∗ Indicates a statistically significant difference (p < 0.01) (Fisher's exact tests).

Our results reveal that very early-onset (<5 years old) and syndromic cases are particularly enriched for monogenic etiologies, with a diagnostic yield of 33% and 40% respectively. Amongst positive genetic diagnoses, very early-onset and syndromic lupus represented 29 and 35% (Table 2). By contrast, family history alone does not appear to be associated with a high rate of positive findings.

## Discussion

We performed exome sequencing on 172 probands with disease onset before the age of 18, along with available family members, totalling 263 individuals. A definitive molecular diagnosis was established in 17 unrelated cases (10%), based on pathogenic or likely pathogenic variants consistent with the phenotype. The diagnostic yield reached more than a third in patients with disease onset before the age of 5 or syndromic features. Unlike adult SLE cohorts where monogenic forms are exceptionally rare and GWAS studies highlight polygenic risk loci, our juvenile cohort demonstrates a 10% monogenic diagnosis rate, underscoring distinct genetic architectures between pediatric and adult-onset disease.

Similar findings have been reported in other cohorts. One study identified a diagnostic yield of 33% in patients with very early-onset (<6 years) lupus, based on exome sequencing of 15 patients.^53^ A study of 72 patients with very early-onset familial, syndromic or aggressive diseases reported a diagnostic yield of 23%, likely reflecting the impact of strict selection criteria disease.^54^ Family history was not associated with a high rate of positive genetic findings in our cohort, in contrast to another study reporting a diagnostic yield of approximately 35% in 35 families. This difference may be explained, at least in part, by the high rate of consanguinity in the population examined in that study.^55^ When broader inclusion criteria were applied, the diagnostic yield decreased. For example, in a cohort of 72 patients with jSLE, predicted damaging variants in 36 known lupus-causing genes were identified in 13% of cases.^56^ Another study involving 50 trios with jSLE led to the identification of a molecular diagnosis in 2 cases (4%), although approximately one-third of patients carried novel or rare variants in genes well established to cause monogenic SLE.^57^ Furthermore, several lupus cohort studies demonstrated an enrichment of rare, predicted deleterious variants in known lupus risk genes, even when these do not correspond to bona fide monogenic forms.^58^^,^^59^ These findings highlight the potential contribution of oligogenic inheritance and underscore the need for further large-scale studies before such approaches can be implemented in routine diagnostic practice.

Our study, the largest exome-sequencing investigation of paediatric onset SLE to date, provides compelling evidence that jSLE is not a single disease entity but rather a spectrum of genetically and mechanistically distinct disorders unified by a common clinical phenotype of systemic autoimmunity. This work provides evidence that early-onset SLE should benefit from a genome-wide approach for Mendelian disease identification. Indeed, we identified several molecular diagnoses not previously associated with SLE and variants in four genes absent from our three *in silico* panels (*DOCK11, PTPN2, MAN1B1, ETV6*), illustrating that more monogenic causes of SLE remain to be identified. In cases with syndromic presentation, a pangenomic analysis is especially appropriate for uncovering a genetic cause that may explain at least part of the clinical presentation. Moreover, this approach is also particularly important given the rapid pace of new gene discovery. We identified three novel genes (*SOCS1*, *PTPN2*, and *DOCK11*) associated with SLE, which underscores groundbreaking insights into the disease. These findings highlight the importance of JAK-STAT signalling regulation, that was disrupted by *SOCS1* or *PTPN2* haploinsufficiency and the essential role of cytoskeleton integrity in immune cell mobility and morphology as shown by *DOCK11* deficiency. Homoeostasis of the NF-κB pathway appears to be critical to prevent the development of lupus-like autoimmunity, as illustrated by the *RELA* variant by the lupus phenotype reported in several patients with A20 haploinsufficiency.^60^ Less anticipated is the previously reported mutation of *PSTPIP1*, known to be associated with PAMI syndrome, which here presented with a lupus phenotype. These cases underscore the possibility of a dual phenotype, autoinflammatory and/or autoimmunity, arising from pathogenic variants, beyond Type I interferonopathies, highlighting innate immunity defect as leading causes of monogenic lupus. In addition, these discoveries significantly advance our understanding of SLE, adding to recent findings that showed the critical role of the regulation of nucleic metabolism and the TLR7 pathway's sensing and signalling mechanisms. Among the unexpected diagnoses, we identified pathogenic variants in genes associated with other PIDD, which are rarely or never linked to SLE, suggesting that a broad targeted panel approach could be a cost-effective alternative.

Variants in *ETV6* have not previously been associated with SLE or immune dysregulation. However, a recent publication suggested that ETV6 may play a role in the transcriptional repression of interferon-stimulated genes, providing a potential mechanistic link to SLE pathogenesis.^61^ Additionally, autoimmune manifestations are well documented in other platelet disorders, such as Wiskott-Aldrich syndrome or grey platelet syndrome.^62^^,^^63^ Notably, the observation of an autoimmune phenotype in mice lacking the Wiskott-Aldrich syndrome protein (WASP) specifically in platelets supports a central role for platelets in immune regulation.^64^ Taken together, the patient's genotype likely explains the thrombocytopaenia, and may also represent a risk factor for the development of autoimmunity.

Similarly, the patient's biallelic *MAN1B1* pathogenic variants aligns well with the major features of MAN1B1-CGD, a congenital disorder of glycosylation characterised by neurodevelopmental delay, truncal obesity, and early puberty. While *MAN2B2* has recently been linked to SLE phenotype,^65^ and growing evidence supports the role of protein glycosylation in innate immune sensing and unfolded protein response, autoimmunity has not been reported in MAN1B1-CDG. Nonetheless, immunologic phenotypes, particularly immunodeficiencies, have been described in other CDG syndromes.^66^

Additionally, the diagnostic yield could be further enhanced by trio genome sequencing, which improves the detection of CNVs and intronic variants. Our reported diagnostic yield is likely underestimated due to several study limitations, including the intrinsic constraints of exome sequencing, the absence of comprehensive CNV analysis, and the limited capacity to perform segregation studies in most cases due to the predominance of singleton exomes. Moreover, the diagnostic yield may be higher in the future as novel disease-associated genes continue yet to be discovered.

In conclusion, we demonstrated that jSLE is monogenic in at least 10% of cases using an exome-sequencing approach. This diagnosis rate is likely conservative, as many disease-associated genes remain to be discovered, and future technological advances will further enhance detection capabilities. Importantly, since targeted therapies can be guided by the genetic diagnosis, our findings strongly support the integration of comprehensive genetic testing into the standard of care in jSLE, with the potential to transform both clinical management and our understanding of disease biology.

## Contributors

MT, FRL, and AB conceived the study; planned, designed and, interpreted experiments. MT wrote the initial draft. ALab, GL, DS performed exome sequencing and participated in variant analysis with QR, TS, CM, and TW. ALab and MT did the initial filtering of variants and the dataset. LH did transcript analysis of *RELA* variant. TM, MY made the functional testing of *IKZF3* variant. SKP made the figures and provided support to the collection of clinical data and biosamples. ALM, QR, MT made functional testing of *SOCS1* and *PTPTN2* variants. SV performed the IFN signatures. Complement dosage was done by VFB, CES and structural 3D structures of C1Q was done by NT and CG. Autoantibody screening was done by NF and DG. Biobanking and processing of samples were done by EC and IR. NP, RL, BR, ALau, MFD, MJ, AM, EH, IM, IKP, CB, CR, HR, PP, JH, ID, OR, YH, FL, JCL, ID, AWM, CP, WM, FSR, BBM provided clinical samples and critically reviewed patient data. AB and MT had full access to all data in the study and take responsibility for the integrity and accuracy of the data analysis. ALM and QR accessed and verified the underlying genetic and bioinformatics data. SKP and AB accessed and verified the clinical data and biosamples. GENIAL/LUMUGENE collaborators provided additional samples. All authors read, critically reviewed and approved the final manuscript.

## Data sharing statement

The data that support the findings of this study are not publicly available because they contain information that could compromise participant privacy. Deidentified data may be available from the corresponding author on reasonable request and with appropriate institutional approvals.

## Declaration of interests

AB reports consulting fees from Novartis, AstraZeneca, Pfizer, GSK, outside the submitted work. IKP reports consulting fees from Novartis, Sobi, Abbvie, Amgen, Chugai outside the submitted work and participated to advisory board for OTEZLA studies. TM reports consulting fees from Astellas Pharma, Takeda Pharmaceuticals, CSL Behring outside the submitted work. JH reports consulting fees from Alnylam Pharma and Bayer and honoraria for lecture from Sanofi outside the submitted work, he participated to the Data Safety Monitoring Board or advisory board from Obitins Trial and is Council member of ESPN. ID reports travelling grant from Mylan and Zambon. CR reports grants from Biogen, Nordic Pharma, Lilly to his institution and consulting fees from Abbvie, Alpha Sigma, Astrazeneca, Boehringer Ingelheim, GSK, Novartis, Pfizer, Zenas Bio outside the submitted work, travelling grant from Abbvie, Novartis and Pfizer and grant support from Biogen, Nordic Pharma and Lilly. All other authors, including GENIAL/LUMUGENE consortium, declare no competing interests.

## Appendix A Supplementary data

Supplementary Tables

Collab Authors
