# Supplementary material for: Exome sequencing enables molecular diagnosis in 10% of early-onset or familial systemic lupus erythematosus cases
Source: eBioMedicine. 2026 Apr 1;126:106209. doi: 10.1016/j.ebiom.2026.106209 (PMC13069521; doi:10.1016/j.ebiom.2026.106209)
Supplement: Supplementary Methods [file mmc1.docx]

***Supplementary methods***

***In silico panel 1***

Monogenic lupus causing genes_2025

| Gene | Reference |
| --- | --- |
| *C1QA* | (Botto et al., 1998; Martens et al., 2009) |
| *C1QB* | (Botto et al., 1998; Martens et al., 2009) |
| *C1QC* | (Botto et al., 1998; Martens et al., 2009) |
| *C1R* | (Lee et al., 1978) |
| *C1S* | (Amano et al., 2008; Dragon-Durey et al., 2001; Lee et al., 1978) |
| *C2* | (Agnello et al., 1972; Stern et al., 1976) |
| *C3* | (Tsukamoto et al., 2005) |
| *C4A* | (Schaller et al., 1977; Paul et al., 2002; Yang et al., 2007) |
| *C4B* | (Schaller et al., 1977; Paul et al., 2002; Yang et al., 2007) |
| *C5* | (Asghar et al., 1991) |
| *C6* | (Trapp et al., 1987 ; Dragon-durey et al., 2003) |
| *C7* | (Segurado et al., 1992) |
| *C8A* | (Jasin, 1977) |
| *C8G* | (Jasin, 1977) |
| *C8B* | (Jasin, 1977) |
| *C9* | (Kawai et al., 1989) |
| *SERPING1* | (Triggianese et al., 2024) |
| *DNASE1* | (Yasutomo et al., 2001) |
| *DNASE1L3* | (Al-Mayouf et al., 2011) |
| *TNK2* | (Guillet et al., 2024) |
| *PTK6* | (Guillet et al., 2024) |
| *ADAR* | (Crow et al., 2015; Linvingston et al., 2016) |
| *RNASEH2A* | (Crow et al., 2015; Linvingston et al., 2016) |
| *RNASEH2B* | (Crow et al., 2015; Linvingston et al., 2016) |
| *RNASEH2C* | (Crow et al., 2015; Linvingston et al., 2016) |
| *TREX1* | (Lee-Kirsch et al., 2007; Crow et al., 2015; Linvingston et al., 2016; Namjou et al., 2011) |
| *SAMHD1* | (Crow et al., 2015; Linvingston et al., 2016; Ramantani et al.,2011) |
| *RIGI* | (Funabiki et al., 2015, Van Eyck et al., 2015) |
| *IFIH1* | (Crow et al., 2015; Linvingston et al., 2016) |
| [*RNU7-1*](https://www.genenames.org/tools/search/#!/genes?query=RNU7-1) |  |
| *LSM11* |  |
| *COPA* | (Watkin et al., 2015) |
| *STING1* | (Jeremiah et al., 2014; Liu et al., 2014; Köning et al., 2017) |
| *POMP* |  |
| *PSMA3* |  |
| *PSMB4* |  |
| *PSMB8* |  |
| *PSMB9* |  |
| *PSMA5* |  |
| *PSMG2* |  |
| *PSMB10* |  |
| *BLM* | (Gratia et al., 2019) |
| *ATM* | (Härtlova et al., 2015) |
| *DCLRE1C* | (Gul et al., 2018) |
| *POLA1* | (Starokadomskyy et al., 2016) |
| *USP18* | (Meuwissen et al.,2016) |
| *ISG15* | (Hermann et  Bogunovic, 2017) |
| *STAT2 (seulement AA R148 et A219)* | (Duncan et al., 2019 ; Gruber et al., 2020) |
| *ACP5* | (Briggs et al., 2011; Bilginer et al., 2016) |
| *DNASE2* | (Rodero et al., 2017 ; Basu et al., 2003) |
| *ATAD3A* | (Lepelley et al., 2021) |
| *PTPN1* |  |
| *ARF1* | (Hirschenberger et al., 2023) |
| *NGLY1* |  |
| *SKIV2L* |  |
| *PNPT1* | (Dhir et al., 2019) |
| *RNASET2* |  |
| *FAS* | (Adachi et al., 1993; Vaishnaw et al. 1999) |
| *FASLG* | (Takahashi et al., 1994; Wu et al 1996) |
| *PRKCD* | (Belot et al., 2013) |
| *PIK3CD* | (Wang et al., 2020 ; Ying et al., 2022) |
| *BRAF* | (Bader-Meunier et al., 2013) |
| *CBL* | (Bader-Meunier et al., 2013) |
| *KRAS* | (Leventopoulos et al., 2014, Zhang et al., 2025) |
| *NRAS* | (Bader-Meunier et al., 2013, Zhang et al., 2025) |
| *PTPN11* | (Quaio et al., 2012, Zhang et al., 2025) |
| *RAF1* | (Bader-Meunier et al., 2013) |
| *SHOC2* | (Bader-Meunier et al., 2013, Morán-Álvarez et al., 2024) |
| *SOS1* | (Bader-Meunier et al., 2013) |
| *ARAF* | (Zhang et al., 2025) |
| *IKZF1* | (Hoshino et al., 2017 . Kuehn et al., 2020) |
| *IKZF2* | (Shahin et al., 2022) |
| *IKZF3* | (Kuehn et al., 2024) |
| *RAG1* | (Chen et al. JACI, 2014) |
| *RAG2* | (Walter et al. 2015) |
| *P2RY8* | (He et al., 2022) |
| *SH2B3* | (Zhang et al., 2024) |
| *SOCS1* | (Hadjhadj et al.,) |
| *PTPN2* | (Jean Pierre et al. 2024) |
| *STAT1* | (Okada et al., 2021) |
| *STAT2* | Duncan et al. (2019) |
| *STAT3* | (Goel et al., 2021) |
| *STAT4* | (Baghdassarian et al., 2023) |
| *STAT5A* | NA |
| *STAT5B* | (A Ma et al., 2017) |
| *STAT6* |  |
| *JAK1* | (Del Bel et al., 2017) |
| *JAK2* |  |
| *JAK3* |  |
| *TLR7* | (Brown, et al., 2022) |
| *UNC93B1* | (Wolf et al., 2024) |
| *TLR3* |  |
| *TLR8* |  |
| *PACSIN1* | (Xie et al., 2023) |
| *CYBA* | (De Ravin et al., 2008; Battersby et al., 2013) |
| *CYBB* | (De Ravin et al., 2008; Battersby et al., 2013) |
| *NCF1* | (De Ravin et al., 2008; Battersby et al., 2013) |
| *NCF2* | (De Ravin et al., 2008; Battersby et al., 2013) |
| *NCF4* | (De Ravin et al., 2008; Battersby et al., 2013) |
| *DOCK8* | (Jouhadi et al., 2014) |
| *DOCK11* | (Boussard et al., 2023) |
| *IRAK4* | (Corray et al, 2024) |
| *BACH2* | (Zhou et al., 2023) |
| *ADA2* | (Schepp et al., 2016) |
| *TNFAIP3* | (Aeschlimann et al., 2017) + Duan 2019 PMID: 31625129 |
| *NLRC4* | (Wang et al., 2023) |
| *PSTPIP1* | (Su et al., 2021) |
| *MAN2B1* | (Urushihara et el., 2004 . Tirosh et al., 2019) |
| *PEPD* | https://www.ncbi.nlm.nih.gov/books/NBK299584/ |
| *SLC7A7* | (Aoki et al., 2001) |
| *MAN1B1* | (Tusseau et al., 2025) |
| *SAT1* | (Xu et al, 2022) |

***In silico panel 2***

Genes from the 2022 Update of IUIS Phenotypical Classification for Human Inborn Errors of Immunity

***In silico panel 3***

Putative lupus causing genes

| **Gene** | **Type of study** | **References** |
| --- | --- | --- |
| *CCDC22* | Case control study | (D'Amico et al., 2017) |
| [*AHNAK2*](https://www.genenames.org/cgi-bin/gene_symbol_report?match=AHNAK2) | EWAS | (Wen et al., 2018) |
| *LCT* | EWAS | (Wen et al., 2018) |
| *TPCN2* | EWAS | (Wen et al., 2018) |
| *ANKRD50* | Familial study | (Delgado-Vega et al., 2018) |
| *CHD3* | Familial study | (Delgado-Vega et al., 2018) |
| *CLC* | Familial study | (Delgado-Vega et al., 2018) |
| *EMC10* | Familial study | (Delgado-Vega et al., 2018) |
| *FAM71E1* | Familial study | (Delgado-Vega et al., 2018) |
| *FAM8A1* | Familial study | (Delgado-Vega et al., 2018) |
| *FBXL14* | Familial study | (Delgado-Vega et al., 2018) |
| *KIR2DS4* | Familial study | (Delgado-Vega et al., 2018) |
| *KRTAP4-9* | Familial study | (Delgado-Vega et al., 2018) |
| *MPHOSPH8* | Familial study | (Delgado-Vega et al., 2018) |
| *NOTCH1* | Familial study | (Delgado-Vega et al., 2018) |
| *NUP214* | Familial study | (Delgado-Vega et al., 2018) |
| *PABPC3* | Familial study | (Delgado-Vega et al., 2018) |
| *PDHA2* | Familial study | (Delgado-Vega et al., 2018) |
| *SCL25A9* | Familial study | (Delgado-Vega et al., 2018) |
| *TRPA1* | Familial study | (Delgado-Vega et al., 2018) |
| *WDR25* | Familial study | (Delgado-Vega et al., 2018) |
| *XRCC6BP1* | Familial study | (Delgado-Vega et al., 2018) |
| *ABHD6* | GWAS | (Bentham et al., 2015) |
| *AFF1* | GWAS | (Okada et al., 2012) |
| *ANKRD44* | GWAS | (Kariuki et al., 2015) |
| *ANKS1A* | GWAS | (Molineros et al., 2017) |
| *ANXA6* | GWAS | (Jing Zhang et al., 2015) |
| *ARHGAP27* | GWAS | (Julià et al., 2018) |
| *ARID5B* | GWAS | (Bentham et al., 2015) |
| *ASPH* | GWAS | (Kariuki et al., 2015) |
| *ATF6B* | GWAS | (Lei et al., 2015) |
| *ATG16L2* | GWAS | (Molineros et al., 2017) |
| *ATG5* | GWAS | (Bentham et al., 2015; Harley et al., 2008) |
| *BANK1* | GWAS | (Kozyrev et al., 2008. Bentham, Nat Genet, 2015) |
| *BC040734* | GWAS | (Alarcón-Riquelme et al., 2016) |
| *C8ORF12* | GWAS | (Harley et al., 2008) |
| *CCL22* | GWAS | (Molineros et al., 2017) |
| *CD226* | GWAS | (Sun et al., 2016; Wang et al.,2018) |
| *CD44* | GWAS | (Lessard et al., 2011; Bentham, Nat Genet, 2015) |
| *CDKN1A* | GWAS | (Harley et al., 2008) |
| *CHAF1B* | GWAS | (Saeed et al., 2017) |
| *CHIA* | GWAS | (Kariuki et al., 2015) |
| *CLEC16A* | GWAS | (Zhang et al., 2011) |
| *COG6* | GWAS | (Márquez et al., 2017° |
| *CRP* | GWAS | (Edberg et al., 2008) |
| *CSK* | GWAS | (Bentham et al., 2015) |
| *CSNK2A2* | GWAS | (Wang et al., 2018) |
| *CXorf21* | GWAS | (Bentham et al., 2015) |
| *DHCR7* | GWAS | (Bentham et al., 2015) |
| [*DLX5*](https://www.genenames.org/cgi-bin/gene_symbol_report?match=DLX5) | GWAS | (Kariuki et al., 2015) |
| *DNAJB3* | GWAS | (Saeed et al., 2017) |
| *ELF1* | GWAS | (Yang et al., 2007) |
| *ETS1* | GWAS | (Bentham et al., 2015; Han et al., 2009; Yang et al., 2010) |
| *FCGR2A* | GWAS | ((Harley et al., 2008; Bentham et al., 2015) |
| *FCGR2B* | GWAS | (Bolland and Ravetch, 2000; Takai et al., 1996; Bentham et al., 2015) |
| *FCGR3B* | GWAS | (Bentham et al., 2015; Fanciulli et al., 2007) |
| *FGF18* | GWAS | (Kariuki et al., 2015) |
| *FLII* | GWAS | (Bentham et al., 2015) |
| *FYB* | GWAS | (Addobbati et al., 2013) |
| *GPR173* | GWAS | (Huoru Zhan et am., 2018) |
| *GPR78* | GWAS | (Liu et al., 2018) |
| *GRB2* | GWAS | (Julià et al., 2018) |
| *GRIN2B* | GWAS | (Saeed et al., 2017) |
| *GRXCR1* | GWAS | (Kariuki et al., 2015) |
| *GTF2I* | GWAS | (Morris et al., 2016; Sun et al., 2016) |
| *GTF2IRD1* | GWAS | (Morris et al., 2016; Sun et al., 2016) |
| *HIST1H3I* | GWAS | (Lei et al., 2015) |
| *ICA1* | GWAS | (Harley et al., 2008) |
| *IKBKE* | GWAS | (Wang et al., 2013; Morris, Nat Genet, 2016) |
| *IKZF2* | GWAS | (Bentham et al., 2015) |
| *IKZF3* | GWAS | (Bentham et al., 2015; Lessard et al., 2012; Wang et al., 1998) |
| *IL12A* | GWAS | (Bentham et al., 2015) |
| *IL4* | GWAS | (Erb et al., 1997; Wu et al., 2003; Yu et al., 2010) |
| *IRF5* | GWAS | (Alarcón-Riquelme et al., 2016; Bentham et al., 2015; Sigurdsson et al., 2005) |
| *ITGAM* | GWAS | (Harley et al., 2008; Nath et al., 2008, Alarcón-Riquelme et al., 2016; Bentham et al., 2015) |
| *ITPR3* | GWAS | (Oishi et al., 2008) |
| *JAZF1* | GWAS | (Alarcón-Riquelme et al., 2016; Bentham et al., 2015)(Gateva et al., 2009) |
| *KCNA5* | GWAS | (Kariuki et al., 2015) |
| *KDM4C* | GWAS | (Alarcón-Riquelme et al., 2016) |
| *KIT* | GWAS | (Liu et al., 2018) |
| *KLK1* | GWAS | (Liu et al., 2009) |
| *LAT2* | GWAS | (Julià et al., 2018) |
| *LATS2* | GWAS | (Saeed et al., 2017) |
| *LBH* | GWAS | (Yu et al., 2013. Morris, Nat Genet, 2016) |
| *LRRC18* | GWAS | (Yang et al., 2010) |
| *LTA* | GWAS | (Lei et al., 2015) |
| *LYN* | GWAS | (Harley et al., 2008; Hibbs et al., 1995) |
| *MBL2* | GWAS | (Font et al., 2007) |
| *MFHAS1* | GWAS | (Wang et al., 2018) |
| *MIR146A* | GWAS | (Bentham et al., 2015) |
| *MON2* | GWAS | (Saeed et al., 2017) |
| *MYNN* | GWAS | (Molineros et al., 2017) |
| *NAA10* | GWAS | (Zhang et al., 2015) |
| *NADSYN1* | GWAS | (Bentham et al., 2015) |
| *NBDY* | GWAS | (Zhu et al., 2015) |
| *NCOA3* | GWAS | (Saeed et al., 2017) |
| *NELFE* | GWAS | (Lei et al., 2015) |
| *NRG3* | GWAS | (Kariuki et al., 2015) |
| *PBX2* | GWAS | (Lei et al., 2015) |
| *PCNXL3* | GWAS | (Alarcón-Riquelme et al., 2016 ; Sun et al., 2016) |
| *PDCD1* | GWAS | (Nishimura et al., 1999; Prokunina et al., 2002) |
| *PDHX* | GWAS | (Lessard et al., 2011) |
| *PHRF1* | GWAS | (Harley et al., 2008) |
| *PLCB4* | GWAS | (Kariuki et al., 2015) |
| *PLD2* | GWAS | (Bentham et al., 2015) |
| *PLEKHF2* | GWAS | (Kariuki et al., 2015) |
| *POU5F1* | GWAS | (Lei et al., 2015) |
| *PRDM1* | GWAS | (Bentham et al., 2015)(Gateva et al., 2009) |
| *PRKCB* | GWAS | (Sheng et al., 2011) |
| *PRKG1* | GWAS | (Kariuki et al., 2015) |
| *PRPS2* | GWAS | (Alarcón-Riquelme et al., 2016; Yan Zhang et al., 2015) |
| *PTTG1* | GWAS | (Harley et al., 2008) |
| *PXK* | GWAS | (Bentham et al., 2015; Harley et al., 2008) |
| *RABGAP1L* | GWAS | (Alarcón-Riquelme et al., 2016) |
| *RAD51B* | GWAS | (Bentham et al., 2015) |
| *RASGRP3* | GWAS | (Han et al., 2009) |
| *RBFOX1* | GWAS | (Kariuki et al., 2015; Saeed et al., 2017) |
| *RNF5* | GWAS | (Lei et al., 2015) |
| [*SCUBE1*](https://www.genenames.org/cgi-bin/gene_symbol_report?match=SCUBE1) | GWAS | (Harley et al., 2008) |
| *SELP* | GWAS | (Jacob et al., 2007) |
| *SH2B3* | GWAS | (Bentham et al., 2015) |
| *SIGLEC6* | GWAS | (Sun et al., 2016) |
| *SKP1* | GWAS | (Bentham et al., 2015) |
| *SLAMF6* | GWAS | (Kumar et al., 2006; Morel et al., 2001; Wandstrat et al., 2004) |
| *SLC12A1* | GWAS | (Alarcón-Riquelme et al., 2016) |
| *SLC15A4* | GWAS | (Han et al., 2009) |
| *SLC22A12* | GWAS | (Alarcón-Riquelme et al., 2016) |
| *SMG7* | GWAS | (Bentham et al., 2015; Deng et al., 2017) |
| *SMYD3* | GWAS | (Julià et al., 2018) |
| *SNX6* | GWAS | (Saeed et al., 2017) |
| *SOCS1* | GWAS | (Bentham et al., 2015) |
| *SPRED2* | GWAS | (Bentham et al., 2015) |
| [*ST3GAL4*](https://www.genenames.org/cgi-bin/gene_symbol_report?match=ST3GAL4) | GWAS | (Wang et al., 2018) |
| *ST8SIA4* | GWAS | (Julià et al., 2018) |
| *STAT4* | GWAS | (Remmers at al., 2007) |
| *SYNGR1* | GWAS | (Sun et al., 2016) |
| *TCF7* | GWAS | (Bentham et al., 2015; Sun et al., 2019) |
| *TMEM187* | GWAS | (Zhang et al., 2015) |
| *TMEM39A* | GWAS | (Lessard et al., 2012) |
| *TMPRSS5* | GWAS | (Kariuki et al., 2015) |
| *TNFAIP6* | GWAS | (Saeed et al., 2017) |
| *TNFSF14* | GWAS | (Wang et al., 2001) |
| *TNFSF4* | GWAS | (Cunninghame Graham et al., 2008), Bentham, Nat Genet, 2015 |
| *TNIP1* | GWAS | (Alarcón-Riquelme et al., 2016; Bentham et al., 2015; Gateva et al., 2009; Han et al., 2009) |
| *TNPO3* | GWAS | (Alarcón-Riquelme et al., 2016) |
| *TNXB* | GWAS | (Kamatani et al., 2008) |
| *TRAPPC11* | GWAS | (Liu et al., 2018) |
| *TTF1* | GWAS | (Saeed et al., 2017) |
| *UBE2L3* | GWAS | (Bentham et al., 2015; Harley et al., 2008) |
| *UHRF1BP1* | GWAS | (Bentham et al., 2015; Gateva et al., 2009) |
| *USMG5* | GWAS | (Alarcón-Riquelme et al., 2016) |
| *WDFY4* | GWAS | (Yang et al., 2010; Alarcón-Riquelme et al., 2016; Bentham et al., 2015) |
| *XKR6* | GWAS | (Budarf et al., 2011) |
| *ZFP90* | GWAS | (Morris et al., 2016) |
| *ZMYND8* | GWAS | (Lei et al., 2015) |
| *ZNF165* | GWAS | (Lei et al., 2015) |
| *ZNRD1* | GWAS | (Lei et al., 2015) |
| *ZSCAN9* | GWAS | (Lei et al., 2015) |
| *BLK* | GWAS; mouse model | (Bentham et al., 2015; Hom et al., 2008; Samuelson et al., 2012) |
| *CIZ1* | GWAS; Mouse model | (Balomenos et al., 2000; Kim et al., 2009) |
| *FLI1* | GWAS; mouse model | (Morris et al., 2010; Zhang et al., 1995) |
| *IFNA17* | GWAS; mouse model | (Li et al., 2005) |
| *MECP2* | GWAS; Mouse model | (Bentham et al., 2015; Sawalha et al., 2008) |
| *NFE2L2* | GWAS; mouse model | (Córdova et al., 2010; Li et al., 2004) |
| *PTPN22* | GWAS; mouse model | (Bentham et al., 2015; Kyogoku et al., 2004; Zikherman et al., 2009) |
| *STRA13* | GWAS; mouse model | (Christensen et al., 2005; Huang et al., 2012) |
| *TLR7* | GWAS; mouse model | (Fairhurst et al., 2008; Pisitkun et al., 2006) |
| *TLR9* | GWAS; mouse model | (Christensen et al., 2005; Huang et al., 2012) |
| *TNFSF13B* | GWAS; mouse model | (Mackay et al., 1999) |
| *APCS* | Mouse model | (Ehrenstein et al., 1998, 2000) |
| [*BCL2*](https://www.genenames.org/cgi-bin/gene_symbol_report?match=BCL2) | Mouse model | (Kozyrev et al., 2008; Liphaus et al., 2006) |
| [*BHLHE40*](https://www.genenames.org/cgi-bin/gene_symbol_report?match=BHLHE40) | Mouse model | (Sun et al., 2001) |
| *CBLB* | Mouse model | (Bachmaier et al., 2000; Yi et al., 2000) |
| *CD22* | Mouse model | (Cornall et al., 1998; O’Keefe et al., 1996) |
| *CD48* | Mouse model | (Kumar et al., 2006; Morel et al., 2001) |
| *CFLAR* | Mouse model | (Qiao et al., 2010; Shenoy et al., 2001) |
| *CSF2* | Mouse model | (Dranoff et al., 1994; Enzler et al., 2003) |
| *DEF6* | Mouse model | (Fanzo et al., 2006; Sun, Nat Genet Letters, 2016) |
| *E2F2* | Mouse model | (Murga et al., 2001) |
| *EP300* | Mouse model | (Forster et al., 2007; Shikama et al., 2003) |
| *GADD45A* | Mouse model | (Hollander et al., 1999; Li et al., 2010) |
| *GPR132* | Mouse model | (Le et al., 2001) |
| *ICMT* | Mouse model | (Doyle et al., 2003) |
| *IFNG* | Mouse model | (Hirankarn et al., 2009; Seery et al., 1997) |
| *IL2* | Mouse model | (Crispín and Tsokos, 2009; Schorle et al., 1991) |
| *IL2RB* | Mouse model | (Lieberman and Tsokos, 2010; Suzuki et al., 1995) |
| *JUNB* | Mouse model | (Meixner et al., 2008; Pflegerl et al., 2009) |
| *LY9* | Mouse model | (Kumar et al., 2006; Morel et al., 2001) |
| *MAN2A1* | Mouse model | (Chui et al., 1997) |
| *MARCO* | Mouse model | (Rogers et al., 2009) |
| *MARK2* | Mouse model | (Hurov et al., 2001) |
| *MERTK* | Mouse model | (Scott et al., 2001; Wu et al., 2011) |
| *MFGE8* | Mouse model | (Hanayama et al., 2004; Hu et al., 2009; Yamaguchi et al., 2008) |
| *MTA2* | Mouse model | (Lu et al., 2008) |
| *NEIL3* | Mouse model | (Massad et al., 2016) |
| *P2RX7* | Mouse model | (Elliott et al., 2005; Portales-Cervantes et al., 2010) |
| *PECAM1* | Mouse model | (Wilkinson et al., 2002) |
| *PPARD* | Mouse model | (Mukundan et al., 2009) |
| *PPARG* | Mouse model | (Oxer et al., 2011; Rosner et al., 2011; Yeh et al., 2008) |
| *PTPN6* | Mouse model | (Green and Shultz, 1975; Shultz et al., 1993; Tsui et al., 1993) |
| *RASSF5* | Mouse model | (Katagiri et al., 2004, 2011) |
| *RC3H1* | Mouse model | (Vinuesa et al., 2005) |
| *RXRA* | Mouse model | (Núñez et al., 2010; Rosner et al., 2011) |
| *SH2D2A* | Mouse model | (Drappa et al., 2003) |
| *SLAMF1* | Mouse model | (Keszei et al., 2011) |
| *TGFB1* | Mouse model | (Dang et al., 1995; Geiser et al., 1993) |
| *TRIM21* | Mouse model | (Espinosa et al., 2009; Frank et al., 1993) |
| *TROVE2* | Mouse model | (Schulte-Pelkum et al., 2009; Xue et al., 2003) |
| *AIM2* | Pathway based study | (Caneparo et al., 2018) |
| *IFI16* | Pathway based study | (Caneparo et al., 2018) |
| *MB21D1* | Pathway based study | (An et al., 2017) |
| *ROBO3* | Pathway based study | (Gao et al., 2015) |
| *IL12RB2* | Targeted gene study | (Kara et al., 2018) |
| *MEF2D* | Targeted gene study | (Farias et al., 2018) |
