# Supplementary material for: Exome sequencing enables molecular diagnosis in 10% of early-onset or familial systemic lupus erythematosus cases
Source: eBioMedicine. 2026 Apr 1;126:106209. doi: 10.1016/j.ebiom.2026.106209 (PMC13069521; doi:10.1016/j.ebiom.2026.106209)
Supplement: Collab Authors [file mmc3.docx]

| **First Name** | **Surname** |
| --- | --- |
| Florence | Aeschlimann |
| Lise | Allard |
| Laurent | Besson-Leaud |
| Mélanie | Blin |
| Karine | Brochard-Payet |
| Antoine | Briantais |
| Guylhène | Bourdat |
| Stéphane | Burtey |
| Alexandra | Cambier |
| Aurélia | Carbasse |
| Wadih | Abou-Chahla |
| Bilade | Cherqaoui |
| Éloïse | Colliou |
| Stéphane | Decramer |
| Véronique | Despert |
| Catherine | Didailler |
| Olivier | Dunard |
| Perrine | Dusser-Benesty |
| Camille | Faudeux |
| Hugues | Flodrops |
| Etienne | Ghrenassia |
| Olivia | Gillion-Boyer |
| Fitsum | Guebre-Egziabher |
| Philippe | Guilpain |
| Vincent | Guigonis |
| Rose-Marie | Herbigneaux |
| Dirk | Holzinger |
| Raju | Khubchandani |
| Charlotte | Kevorkian-Verguet |
| Martin | Killian |
| Noémie Le | Gouellec |
| Irène | Lemelle |
| Iona | Madden |
| Hazem | Manadile |
| François | Maurier |
| Ulrich | Meinzer |
| Sandrine | Morell-Dubois |
| Antoine | Mouche |
| Anne-Sophie | Parentelli |
| Alice | de Pellegars Malhortie |
| Isabelle | Pellier |
| Pierre | Quartier dit Maire |
| Karine | Retornaz |
| Linda | Rossi |
| Caroline | Rousset-Rouvière |
| Adrien | Subervie |
| Soizic | Tiriau |
| Florence | Uettwiller |
| Diego | Urbina |
| Jean-François | Viallard |
| Franck | Zekre |
